# IL-17-producing peripheral blood CD177^+^ neutrophils increase in allergic asthmatic subjects

**DOI:** 10.1186/1710-1492-9-23

**Published:** 2013-07-03

**Authors:** Carlos Ramirez-Velazquez, Elena Cristina Castillo, Leopoldo Guido-Bayardo, Vianney Ortiz-Navarrete

**Affiliations:** 1 Molecular Biomedicine Department, Centro de Investigación y de Estudios Avanzados (CINVESTAV)-IPN, Av. IPN No. 2508, Colonia San Pedro Zacatenco, México, DF CP. 07360, México; 2 Allergy Department, Hospital General Dr. Fernando Quiroz Gutiérrez, ISSSTE, Calle Felipe Angeles y Canario. Colonia Bellavista, Mexico, DF CP 01140, Mexico; 3 Allergy Department, Centro Médico Nacional 20 de Noviembre ISSSTE, Felix Cuevas 540, Colonia del Valle, Mexico, DF CP 03229, Mexico

**Keywords:** Neutrophils, IL-17, Allergic asthma, Blood

## Abstract

**Background:**

A T helper cell (T_H_) 17-biased response has been observed in patients with allergic asthma, particularly in those with neutrophil accumulation in the lung. Therefore, we sought to test the hypothesis that neutrophils might be an important source of interleukin (IL)-17 in allergic asthma.

**Methods:**

Whole peripheral blood cells from non-asthmatic control subjects (n = 17) and patients with mild asthma (n = 7), moderate but persistent asthma (n = 4), or acute asthma (n = 6) were analyzed for IL-17A expression in CD177^+^ neutrophils. IL-17A expression was also analyzed in CD3^+^CD4^+^ and CD3^+^CD8^+^ lymphocyte populations. Asthmatic patients were classified as allergic to fungi, indoor allergens, or other allergens (*e.g.*, pollen) based on a positive intradermal allergy test reaction.

**Results:**

The percentage of CD177^+^ neutrophils in whole blood of asthmatic patients was higher than in healthy controls and highest in the moderate asthma group. Furthermore, the percentage of CD177^+^IL-17^+^ neutrophils was elevated in patients with mild asthma, whereas the CD4^+^ IL-17^+^ lymphocyte population was higher in asthmatic patients and highest in those with moderate but persistent asthma. We also found that the four patients that were allergic to fungi had the highest percentage of CD177^+^IL17^+^ neutrophils and CD8^+^IL17^+^ lymphocytes.

**Conclusion:**

IL17^+^CD177^+^ Neutrophils increase in allergic asthma patients especially when allergic to fungi. This cell population, through release of IL-17, might be contributing during the initial phase asthmatic disease and/or during disease progression but its role has not yet been established.

## Background

Asthma is a heterogeneous chronic inflammatory respiratory disease characterized by overproduction of mucus and airway-wall remodeling that leads to bronchial hyperactivity and airway obstruction [1]. Allergens and some pathogens have been implicated in the worsening of asthma [2,3], and the disease can be classified as mild, moderate, or severe according to the magnitude of the inflammation [4].

For many years, allergic asthma has been considered a T helper 2 (T_H_2)-biased disease, characterized by eosinophil infiltration and the production of the cytokines interleukin (IL)-4, IL-5, and IL-13 [5]. A T_H_17-biased response has also been observed in patients that exhibit chronic inflammation [6] and particularly in those with severe asthma who respond poorly to steroids, where inflammatory cellular infiltration in the airway is primarily due to CD4^+^ T_H_17 cells and neutrophils [7-9].

Neutrophils have been associated with the severity of asthma [10,11]. Moreover, studies in humans have demonstrated neutrophil recruitment in response to allergen challenge that coincides with the peak of CD4^+^ T-cell recruitment. The peak of eosinophil recruitment occurs several days later [12], suggesting the importance of neutrophils in the pathogenesis of the disease. In addition, it has been reported that IL-17 favors neutrophil recruitment and leads to the induction of neutrophilia rather than eosinophilia in rodents [13,14]. The numbers of neutrophils in the sputum [9,10,15], bronchoalveolar lavage [12], bronchial biopsies [16,17], and peripheral blood [18] of allergic asthmatic patients have been shown to increase concomitantly with IL-17 levels [7,19-21].

IL-17 is mainly produced by T_H_17 cells, but also by CD8^+^ T cells, γδ T cells, natural killer cells, and granulocytes [22]. In addition, it has been shown that murine neutrophils release IL-17 [13,23], but no further studies have investigated the expression and release of IL-17A -the most common form of IL-17- from human peripheral blood neutrophils in neither a normal state or during disease ( *e.g.,* allergic asthma).

In this study, we demonstrated that in fact human neutrophils are able to express IL-17. We also observed increased numbers of IL-17A + neutrophils in peripheral blood of asthmatic patients particularly in those suffering from fungal allergy-associated asthma.

## Methods

### Patients and control subjects

We recruited 17 asthmatic patients and all of them tested positive in the allergen skin prick test (Alerquim, Mexico City) to at least one of following: house dust mites, pollens, and fungi. We classified the asthma severity in our patients according to Global strategy for asthma management and prevention: GINA executive summary 2008 [24]. In our study we only included patients who matched the mild (seven patients) or moderate (four patients) categories according to GINA.

We also included acute asthma patients (six patients) defined as those who show exacerbation in symptoms such as wheezing, breathlessness, and chest tightness 48 hours prior to admission to the emergency department and received only rescue medication. These patients were enrolled within 24 hours of admission to the emergency department. Prior to the start of treatment, a blood sample was obtained for this study. However, three out of the six patients with acute asthma had inhaled β2-agonist short acting bronchodilators 48 h before their hospital admission. Patients who had an infection process along with the exacerbation were not included.

All subjects (asthmatic and control) were either nonsmokers or former smokers who had quit smoking for at least 12 months. Subjects who had used corticosteroids, long-acting β2-agonists, leukotriene antagonists, or antihistamines in the month preceding the study were excluded, so were subjects with history of respiratory tract infection in the 4 weeks preceding the study. Healthy subjects without history of allergy or bronchial symptoms and who tested negative in the allergen skin prick test (Alerquim) made up the control group. Total serum immunoglobulin E was measured in every subject as well as the forced expiratory volume in 1 second (FEV1). Table 1. Three different independent measurements of FEV1 were performed with a dry spirometer (Medgraphics, Minnesota, USA) and the optimum value was expressed as a percentage of the predicted value. The Ethics Committee of the Fernando Quiroz Hospital approved the study, and each subject gave written informed consent.

**Table 1:** Characteristics of study subjects

	**Asthmatics**	**Non-asthmatic controls**
Sex (female/male)	6/11	7/10
Age (y) (mean ± SEM)	22.35 ± 3.82	24.12 ± 1.38
Atopy (Nº)^1^	17/17	0/17
Serum total IgE levels (IU/mL) (mean ± SEM)	425.2 ± 105.2**	278.89 ± 14.6
FEV1 (% predicted)	48-117	88-111
(mean ± SEM)	(77.65% ± 4.77)**	(96.59% ± 1.61)

^1^Atopy is defined as at least one positive prick test.

**P < .01 compared to non-asthmatic controls.

Abbreviations: *FEV1* Forced expiratory volume in 1 second, *Ig* Immunoglobulin, *SEM* Standard error of the mean.

### Preparation of human mononuclear cells

Whole blood cells were obtained from 17 healthy volunteers and 17 asthmatic patients. Peripheral blood mononuclear cells (PBMCs) were isolated using a differential centrifugation gradient (Ficoll-Paque PLUS, GE Healthcare). The PBMCs were analyzed for viability with trypan blue, washed, and grouped into two, either for the *ex-vivo* staining or *in vitro* activation.

### Cell activation

Heparinized whole blood (HWB; 500 μL) was stimulated with 2 μg/mL ionomycin (Sigma-Aldrich) and 40 ng/mL phorbol myristate acetate (PMA; Sigma-Aldrich) for 18 h at 37°C. PBMCs were stimulated with 200 ng/mL ionomycin and 2 ng/mL PMA for 18 hours at 37°C. In both cases 10 μg/mL brefeldin A (BFA; Sigma-Aldrich) was added during the last 6 hours of culture activation.

### Surface staining and intracellular cytokine detection

Cells from activated and non-activated HWB were stained with fluorescein isothiocyanate (FITC)-conjugated anti-CD177 and phycoerythrin (PE)-conjugated anti-IL-5Rα (R&D) for 20 min at 4°C. Blood erythrocytes were lysed with lysis buffer solution (155 mM NH4C1, 10 mM KHCO3, and 0.1 mM EDTA, pH 7.3) for 15 min at room temperature (RT). Subsequently, cells were permeabilized using FACS Perm2 solution (BD Biosciences, San Jose, CA, USA) for 10 min based on manufacturer recommendations. Then samples were stained with peridinin chlorophyll-A protein (PerCP)/Cy5.5 conjugated anti-IL-17A (BioLegend). Finally cells were fixed with 2% paraformaldehyde (PFA) and analyzed using a CyAn ADP cytometer (Beckman Coulter, Inc. Indianapolis, IN; USA). Activated and non-activated PBMCs were stained with FITC-conjugated anti-CD-3, allophycocyanin (APC)-Cy7-conjugated anti-CD4 and PE-conjugated anti-CD8 for 20 min at 4°C and afterwards permeabilized, stained for IL-17A and CD69, and fixed as described above. Cells were washed after fixation and analyzed with a CyAn ADP cytometer (Beckman Coulter). Isotype-control matched mAbs (BioLegend) were used as negative controls for each fluorochrome.

### Flow cytometry analysis

Neutrophils were identified according to size (forward scatter, FSC) and complexity (side scatter, SSC) and by the expression of CD177 (BioLegend). Eosinophils IL-5Rα marker was used to distinguish them from neutrophils in HWB to further determine the percentage of CD177^+^IL17^+^ neutrophils. IL-17 expression was also evaluated in CD3^+^CD4^+^ and CD3^+^CD8^+^ lymphocytes from PBMCs previously gated according to FSC and SSC as well. CD69 was used as an activation marker for T cells which were activated with ionomycin-PMA. Data analysis was performed using the FlowJo 5.6.4. software.

### Statistical analysis

Distributions of continuous variables are expressed as mean ± standard error (SEM) and median. A nonparametric Mann–Whitney U test was used to compare continuous variables, the Wilcoxon test for 2-group comparisons, and the Kruskal-Wallis test for multiple comparisons. The Friedman post hoc test was used to confirm differences in individual groups. *P* values less than .05 were interpreted to indicate significance.

## Results

### Peripheral blood neutrophils express IL-17 and increase in number in allergic asthma patients

**Figure 1:**
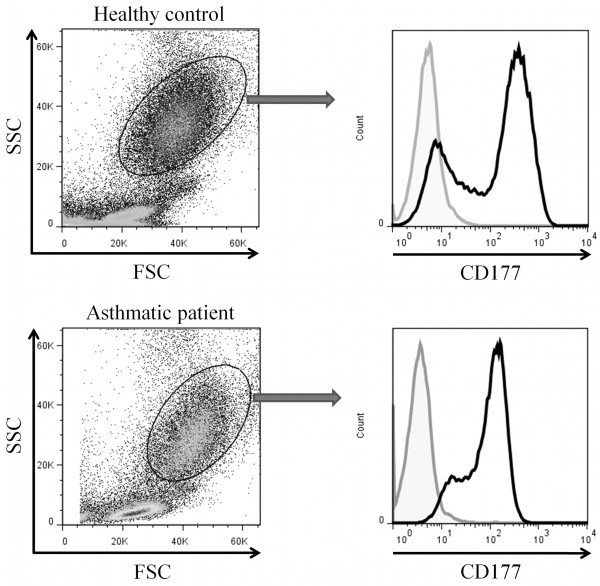
**Peripheral blood neutrophils were identified within the granulocytes cells according to size (forward scatter), complexity (side scatter) and CD177 expression.** A representative dot plot and histogram is shown from one patient and one healthy control.

**Figure 2:**
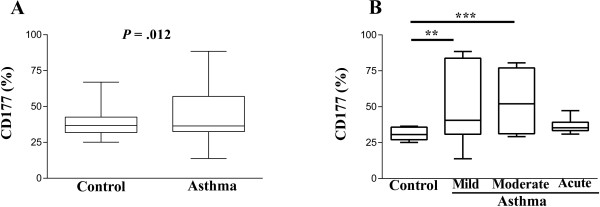
**Neutrophils increase in patients with allergic asthma. (A)** The percentage of CD177^+^ neutrophils in allergic asthma and healthy control subjects is shown. **(B)** Percentages of CD177^+^ neutrophils from patients with mild, moderate, and acute asthma in comparison with control non-asthmatic subjects. The asterisks indicate **P = .01, ***P = .0001. Error bars indicate SEM.

**Figure 3:**
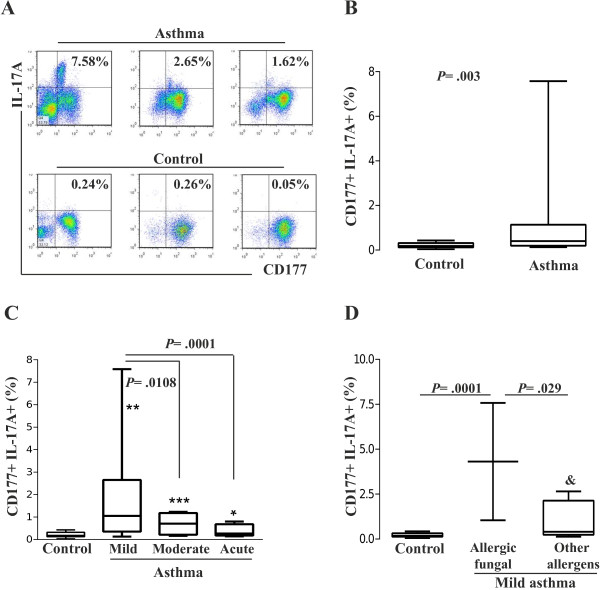
**Neutrophils express IL-17A. (A)** Percentages of CD177^+^ IL-17A^+^ neutrophils from allergic asthma patients and healthy controls. **(B)** Percentages of CD177^+^ cells that are IL-17A^+^. **(C)** Percentage of IL-17A^+^ cells stratified by the severity of allergic asthma and **(D)** by responses to allergy tests in mild asthma patients. The symbols indicate *P = .04, **P = .01, ***P = .005, and &*P* = .001. Error bars indicate SEM., and &*P* = .001.

The neutrophils population in HWB was analyzed by flow cytometry according to size (forward scatter, FSC) and complexity (side scatter, SSC) as well as the expression of CD177 (Figure 1). The percentage of neutrophils in the whole blood of asthmatic patients was higher than in healthy controls (*P* = .012; Figure 2A), and was highest in patients with moderate asthma (*P* = .0001), but no differences were observed between patients with acute asthma and healthy controls (Figure 2B). We observed that the percentage of CD177^+^IL-17^+^ neutrophils in allergic asthmatic patients was higher (*P* = .003) when compared to healthy controls (Figure 3A, 3B). No further increase was observed when neutrophils were stimulated with ionomycin/PMA (data not shown). In addition, we found that the percentage of CD177^+^IL-17^+^ neutrophils increased in mild (*P* = .01), moderate (*P * = .005) and acute asthma (*P* = .041) in comparison to healthy controls. The CD177^+^IL-17^+^ neutrophils percentage was highest in patients diagnosed with mild asthma, as compared to that in those with moderate asthma (*P* = .0108) or acute asthma (*P* = .0001). However, no significant difference was observed between the latter two groups (Figure 3C).

### The percentage of IL-17A^+^ neutrophils in peripheral blood is higher in asthma patients with fungal allergy

We sought to determine if a specific type of allergen preferentially activated the neutrophil response. To address this we analyzed the percentage of CD177^+^ IL-17A^+^ neutrophils from asthmatic patients that are allergic to specific groups of allergens based on positive intradermal allergy test reactions. Patients were divided into two groups: asthmatic patients allergic to fungi (*Penicilium, Rhizopus, Candida albicans, Alternaria alternata,* and *Aspergillus fumigatus*) and asthmatic patients allergic to other allergens such as pollens (*Lolium perenne, Fraxinus, Liquidambar, Pinus, Quercus, Olea europeae, Amaranthus palmeri, Prosopis,* and *Chenopodium album*) and indoor allergens (dust mites, dogs, cats, and cockroaches). We found that asthmatic patients that are allergic to fungi (4 out of 17) had a higher percentage of CD177^+^ IL-17^+^ neutrophils in their peripheral blood compared to patients with allergic asthma that are reactive to other allergens (*P* = .0001; Table 2). Figure 3D shows that patients with mild asthma who are allergic to fungi (2 out of 6) exhibited the highest percentage of CD177^+^ IL-17^+^ neutrophils as compared to those patients with mild asthma who are allergic to other allergens (*P* = .029) and to healthy controls (allergic fungal *P* = .0001, other allergens &*P* = .04). While analyzing the numbers of neutrophils, we observed an increase in both the percentage of cells that express IL-17 and the number of neutrophils (*P* = .006; Table 2). This data suggests a positive association between neutrophils, fungal allergens, and asthma.

**Table 2:** Comparisons of biomarkers in patients allergic to fungal allergens and to other allergens

	**Fungal allergens** **(n = 4)**	**Other allergens (indoor and pollen allergens)** **(n = 13)**	*P *value*
CD177+ IL-17A + (%)	2.465 ± 1.716	0.6938 ± 0.205	0.0001
IL-17+ TCD4+ (%)	0.405 ± 0.233	0.417 ± 0.165	0.3801
IL-17+ TCD8+ (%)	0.277 ± 0.196	0.148 ± 0.042	0.0065
Neutrophil count (mm^3^)	7325 ± 1656	3761 ± 356.0	0.0067
Eosinophil count (mm^3^)	390.8 ± 218.3	693.5 ± 102.7	0.2931
CD4^+^ T cells (mm^3^)	347.8 ± 147.8	507.9 ± 79.41	0.4001
CD8^+^ T cells (mm^3^)	229.9 ± 56.56	363.5 ± 90.93	0.0528
Serum total IgE levels (IU/mL)	496.43 ± 294.0	352.0 ± 81.82	0.0352
FEV1 (% predicted)	83.75 ± 14.21	75.00 ± 4.99	0.1097

Data are shown as mean ± standard errors of the mean.

Abbreviations: *FEV1* Forced expiratory volume in 1 second, *Ig* Immunoglobulin, *IL* Interleukin.

In addition, we analyzed the percentage of IL-17A^+^ T cells stratified according to the two groups of allergens in the asthmatic patients. We found that the percentage of CD8^+^ IL-17^+^ T cells was higher in patients that are allergic to fungi (*P* = .006; Table 2). On the other hand the percentage of CD4^+^ IL-17A^+^ T cells remained unchanged (*P* = .38; Table 2) even between patients allergic to pollens and indoor allergens (data not shown).

### CD4^+^ IL-17^+^ T lymphocytes increase in number according to asthma severity

**Figure 4:**
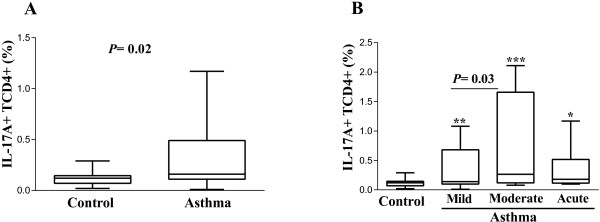
**Percentage of CD4**^**+ **^**T cells expressing IL-17 (Th17) in asthmatic patients. (A)** Comparison of the percentages of Th17 cells from patients and controls. **(B)** Percentage of Th17 cells stratified according to disease severity. Asterisks indicate *P = .04, **P = .007, *** *P* = .0001. Error bars indicate SEM.

PBMCs were isolated from peripheral blood of allergic asthma patients and healthy controls in order to identify IL-17+ CD4^+^ T cells. Analysis was performed using flow cytometry based on the expression of molecular markers by selecting the CD4^+^ IL17A^+^ population by gating for lymphocytes. We observed that the percentage of CD4^+^ IL-17A^+^ T cells was higher in allergic asthma patients than in healthy controls (*P* = .02; Figure 4A). We found that the percentage of CD4^+^ T cells was higher in all groups (mild *P* = .007, moderate *P* = .0001, acute *P* = .04) the highest being in moderate asthma patients (*P* = .03). We found no difference between patients with acute asthma and those with moderate asthma (Figure 4B).

## Discussion

Multiple lines of evidence show a link between an increase in neutrophil numbers and the exacerbation, progression, severity, and difficulties in the control of asthmatic disease [9,10,15], not just at the bronchial level but also in peripheral blood [18]. Some mediators of these cells such as matrix metalloproteinase-9 [25], lactoferrin [26], reactive oxygen species [27], and IL-8 [28] have been associated with poor clinical evolution of the disease. During this study, even though only a small number of patients was analyzed, we found an increase in the percentage of neutrophils in peripheral blood from allergic asthmatic patients. In this same group of patients we observed an IL-17^+^ neutrophil subpopulation increase. The highest increase in this subpopulation was seen in patients with mild asthma. This result suggests that CD177^+^ IL-17A + neutrophils might play a role in the early stages of the response. The difference in the number of IL-17A^+^ neutrophils in the three evaluated groups did not change even after cells were stimulated *in vitro* with ionomycin/PMA (data not shown). This indicates the possible existence of neutrophils that have been polarized to produce IL-17A, as has been described for CD4^+^ IL-17^+^ T cells. However, further studies are required to evaluate whether or not these neutrophils subpopulation express the nuclear hormone receptor RORγt [22]. Recently, Tan Z *et al*., demonstrated that Gr-1^hi^ mouse neutrophils express RORγt and these cells express IL-17A mRNA after stimulation with LPS, LPS/IL-1β, or PMA/ionomycin suggesting that these cells have the capacity to produce IL-17A. The RORγt neutrophils appear to amplify inflammation and damage in ischemia-reperfusion (I/R) injury. Taz Z *et al.* also showed the presence of CD15^hi^ IL-17^+^ neutrophils population in peripheral blood from seven patients who underwent partial hepatectomy. Remarkably these CD15^hi^ neutrophils expressing IL-17A increased upon surgical intervention [29]. However, they are yet to prove whether these cells express RORγt. Their results resemble ours in that there is an IL-17A subpopulation of neutrophils in peripheral blood and that those cells might play a role in pathogenesis thought the release of IL-17A. However, whether this CD15^hi^ IL-17^+^ neutrophil subpopulation is the same as the one described by us is yet to be determined.

We found that patients with fungal allergies (4 of 17) had the highest number of CD177^+^ IL-17^+^ neutrophils. In this regard, Inoue *et al*. reported that the use of β-glucan from *Candida albicans* in the lungs of mice induces neutrophilic airway inflammation and the expression of different cytokines such as IL-17 [30]. Furthermore, Werner *et al*. reported that mouse neutrophils produce IL-17 in a dectin-1-dependent manner following infection with *Aspergillus fumigatus*[31]*.* Thus, we suggest that fungal allergens and their derivatives might activate neutrophils through the dectin-1 receptor, inducing IL-17 production and contributing to bronchial inflammation during allergic asthma.

Additionally, we observed that the numbers of CD8^+^ T IL-17^+^ cells also increased in patients with fungal allergy asthma. Consistent with this observation, it has been reported that neutrophils regulate the infiltration of CD8^+^ T cells to the inflammation site in an animal model for fungal airway allergy [32]. Neutrophils could also act as antigen-presenting cells to promote IL-17 production by CD4 and CD8^+^ T cells [33-35].

Finally, we observed that the percentage of CD4^+^IL-17^+^ T cells in peripheral blood of patients was associated with the severity of asthma, confirming observations described by other groups [6-9,21,36]. We also observed an increase of CD4^+^IL-17^+^ T cells in acute asthma, reinforcing the importance of IL-17A in the immunopathology of asthma. Furthermore, in contrast to neutrophils, the production of IL-17 by CD4^+^ T cells was independent of the type of allergen. Importantly, Pelletier, M *et al.*, described a chemokine-dependent reciprocal cross-talk between human neutrophils and Th17 cells. They described that activated neutrophils induce chemotaxis of Th17 cells by release of CCL2 and CCL20 chemokines. At the same time, CXCL8 is produced by Th17 to chemoattract neutrophils. They also found that CD15^+^ neutrophils and RORγt^+^ cells colocalize in gut tissue from patients with Crohn disease and synovial fluid from rheumatoid arthritis patients [37]. It is likely that the recruitment of neutrophils and Th17 in response to allergen is mediated by the chemokine cross-talk described above.

As it is now known, Th17 response is important for host defense against extracellular bacteria and fungi by indirectly inducing and activating neutrophils through production of IL-17A and IL-17 F. In this context, our study found higher percentage of IL-17^+^ neutrophils in asthma patients who are allergic to fungi. This suggests that this cell subpopulation might be activated by fungal allergens to release IL-17.

## Conclusion

We identified a subpopulation of CD177^+^ IL-17A^+^ neutrophils in peripheral blood of asthmatic patients and healthy controls. Even though we were able to appreciate differences between asthmatic groups, due to the number of analyzed patients, it is not possible to define the relation between this subpopulation and severity of asthma.

## Abbreviations

FEV1: Forced expiratory volume in 1 second; FITC: Fluorescein isothiocyanate; PMA: Phorbol myristate acetate; FACS: Flow cytometry (fluorescence-activated cell sorting); SEM: Standard error of the mean; FSC: Forward scatter; SSC: Side scatter; HWB: Heparinized whole blood; PBMC: Peripheral blood mononuclear cell; IL: Interleukin; TH: T helper.

## Competing interest

The authors declared no conflict of interest.

## Authors’ contributions

CRV and VON designed the experiments; LGB selected the patients; CRV did the experiments; CRV, ECC and VON analyzed the data and wrote the manuscript. All authors read and approve the final manuscript.
